# 530 Fractured Coverage for Fractional Laser: Describing the Insurance Landscape of Laser Therapy for Burn Scars

**DOI:** 10.1093/jbcr/irae036.165

**Published:** 2024-04-17

**Authors:** Tyler R Reinoso, Matthew J Heron, Julia M Dane, Siam K Rezwan, Kristen P Broderick, Julie A Caffrey

**Affiliations:** Drexel University College of Medicine, Philadelphia, PA; Johns Hopkins University School of Medicine, Baltimore, MD; DeBusk College of Osteopathic Medicine, Harrogate, TN; Johns Hopkins University, Baltimore, MD; Drexel University College of Medicine, Philadelphia, PA; Johns Hopkins University School of Medicine, Baltimore, MD; DeBusk College of Osteopathic Medicine, Harrogate, TN; Johns Hopkins University, Baltimore, MD; Drexel University College of Medicine, Philadelphia, PA; Johns Hopkins University School of Medicine, Baltimore, MD; DeBusk College of Osteopathic Medicine, Harrogate, TN; Johns Hopkins University, Baltimore, MD; Drexel University College of Medicine, Philadelphia, PA; Johns Hopkins University School of Medicine, Baltimore, MD; DeBusk College of Osteopathic Medicine, Harrogate, TN; Johns Hopkins University, Baltimore, MD; Drexel University College of Medicine, Philadelphia, PA; Johns Hopkins University School of Medicine, Baltimore, MD; DeBusk College of Osteopathic Medicine, Harrogate, TN; Johns Hopkins University, Baltimore, MD; Drexel University College of Medicine, Philadelphia, PA; Johns Hopkins University School of Medicine, Baltimore, MD; DeBusk College of Osteopathic Medicine, Harrogate, TN; Johns Hopkins University, Baltimore, MD

## Abstract

**Introduction:**

Laser therapy can improve skin texture, range of motion, and cosmesis for patients with burn scars. Nevertheless, insurance coverage for laser therapy is highly variable, leaving patients and providers with unforeseen costs from denied claims. To determine optimal practices for patient documentation and claim reimbursement for laser therapy, we aimed to comprehensively review the insurance policy landscape of this modality for burn scars.

**Methods:**

We identified the largest health insurers by enrollment and market share using data from the National Association of Insurance Commissioners. For each insurer, we identified their policy on laser therapy and conducted a dual, blind extraction of its documentation requirements, prior and continuing authorization requirements, and treatment guidelines. We also collected details on prerequisite failed treatments and suggested treatment alternatives.

**Results:**

We identified 60 health insurance providers. Eleven (18.3%) had explicit policies on scar revision. Nine (15.0%) either had no publicly available policy or could not confirm coverage details. The remaining 40 had general reconstructive surgery policies that were non-specific to burn scars. Nineteen insurers (n = 19) considered laser therapy medically necessary for scar revision, and all policies required documentation of functional impairment to establish medical necessity; however, only nine policies defined functional impairment. Six insurers required patients to fail one or more alternate trials, including “standard therapy” (n = 6), silicone sheeting (n = 5), compression therapy (n = 4), intralesional corticosteroid injections (n = 2), intralesional 5-fluorouracil (5-FU) injections (n = 1), cryotherapy (n = 1), hypoallergenic paper tape (n = 1), or surgical excision (n = 1). Seven health insurers recommended alternatives to laser therapy. Surgical excision was the most recommended alternative (n = 6, 85.7%), followed by intralesional corticosteroid injections (n = 5, 71.4%), superficial radiation therapy (n = 4, 57.1%), silicone sheeting (n = 4, 57.1%), and compression therapy (n = 4, 57.1%). Intralesional 5-FU, cryotherapy, and pulsed-dye laser were recommended less frequently (n = 3 each, 42.9% each). Notably, 3 insurers explicitly denied laser coverage under any circumstance.

**Conclusions:**

Most health insurers have vague policies on laser therapy for burn scar revision, complicating the reimbursement landscape for patients and providers. Burn surgeons can enhance their odds of claim approval by seeking prior authorization, maintaining detailed patient records, and using objective measures for functional impairment and improvement.

**Applicability of Research to Practice:**

This study offers guidance to burn surgeons who use lasers for scar revision and want to maximize their likelihood of insurance reimbursement.

